# Study on botulinum toxin in dermatology from 2000 to 2023: A CiteSpace‐based bibliometric analyses

**DOI:** 10.1111/jocd.16423

**Published:** 2024-07-08

**Authors:** Ziwenyan Zhou, Fujun Huang, Jin Chen

**Affiliations:** ^1^ Department of Dermatology, the First Affilated Hospital of Chongqing Medical University Chongqing China; ^2^ College of Traditional Chinese Medicine, Chongqing Medical University Chongqing China

**Keywords:** bibliometric analysis, botulinum toxin, CiteSpace, dermatology, VOSviewer

## Abstract

**Objective:**

Using bibliometric methods, this study analyzed and summarized the current situation and development of the global application of botulinum toxin in dermatology.

**Methods:**

Literature published in the Web of Science Core Collection database from January 1, 2000 to October 28, 2023 was searched for topics such as “Botulinum toxin,” “Dermatology,” and so forth. The number of publications, countries, institutions, journals, authors, cited literature, keywords, and so forth, were analyzed and a visual knowledge map was created using scientometric tools such as CiteSpace, VOSviewer, and Scimago Graphica.

**Results:**

A total of 2039 documents were retrieved and 1877 documents were included after de‐duplication and transformation. The country with the highest number of published periodical articles was the United States; the main research institution was Yonsei University; the author with the highest number of published periodical articles was Kim, Hee Jin; and the high‐frequency keywords mainly related to indications, combination therapy, and safety optimization.

**Conclusion:**

The results of this study provide information on the current status and trends in clinical studies of botulinum toxin in dermatology, which will help researchers identify hotspots and explore new research directions in this field.

## 1 INTRODUCTION

Botulinum toxin (BTX) is a bacterial exotoxin produced by *Clostridium botulinum*. *C. botulinum* and its toxins are classified into eight types, A, B, C (C1, C2), D, E, F, and G, based on the antigenicity of the toxin. All are neurotoxins except C2, which is a cytotoxin. Of these, botulinum toxin type A (BTX‐A) is the most widely used. The world's first BTX for clinical use is BTX‐A, when the US Food and Drug Administration (FDA) approved the marketing of BTX‐A, in December 1989. When using BTX, be sure to go to a proper organization, and choose a product that complies with national regulations, as BTX is a toxic protein. Otherwise, it may have adverse reactions after use. In dermatology, BTX is now widely used for wrinkles, muscle hypertrophy, acne, scarring, alopecia, osmidrosis, and so forth. In addition, BTX is also widely used in neurology, rehabilitation, urology, and other clinical areas.^1^, ^2^, ^3^, ^4^, ^5^ Meanwhile, the information shows that the global BTX market size reached $360 million in 2016, and is expected to grow year by year at a compound annual growth rate of 9%, which is of great commercial value. Therefore, understanding the hotspots and focuses of BTX is of great importance for the clinical application of BTX.^6^

With the explosive growth in the research literature production, the need for new approaches to structure knowledge emerged. The capability to analyze vast amounts of publications on macroscopic and microscopic levels, together with domain independence classifies bibliometrics as one of such approaches. Hawkins defines it as “a quantitative analysis of the bibliographic characteristics of a large number of documents.”^7^ Bibliometric mapping is one of the bibliometrics tools that uses text mining, co‐word analysis, and clustering algorithms to capture associations between pairs of units of analysis (words, phrases, country, institution or author names, or other terms found in scientific documents) and visualizes them in the form of bibliometric maps.^8^

CiteSpace is a software that tracks and analyzes international cutting‐edge research, which is used to illustrate the development of a discipline or knowledge field and track research hotspots and frontiers.^9^ VOSviewer is a software for building and visualizing co‐occurrence networks of keywords extracted from a large body of scientific literature. The Web of Science Core Collection database (WoSCC) is of excellent quality and covers most of BTX's high‐quality research in the field of dermatology.

Therefore, we integrated and performed a bibliometric analysis of the literature published in WoSCC from 2000 to 2023 on the application of BTX in dermatological research. It aims to summarize the scope of application of BTX in dermatology and its indications, to explore the scope of application, to research hotspots, to predict future research trends, and to formulate more effective and efficient treatment protocols. The findings of this article will provide a reference for clinicians and researchers in this field.

## 2 METHOD

The main two advantages of bibliometrics are domain independence and that it enables analyses of large quantities of publications. Bibliometric mapping visualizes and maps the relationships and associations between units (terms, authors, source titles, countries, and similar topics) into a research landscape using text mining and clustering algorithms. A bibliometric map is a network of nodes where nodes represent units and the links between nodes represent relationships. The proximity of nodes can be interpreted as an indicator of their similarity, while the node size might indicate the popularity of a unit. Clusters of nodes, usually indicated by different colors, represent units that are strongly associated.^8^, ^10^

The source of the study was WoSCC, and the search strategy and retrieval process are shown in Figure 1. CiteSpace (5.7.R5) was applied to perform bibliometric analysis, and knowledge maps of countries/regions, institutions, journals, authors, keywords, and references were generated. The retrieved articles were exported in “Plan text file” and “Full Record and Cited References” formats, and then imported into CiteSpace for further analysis.^9^, ^11^

**FIGURE 1:**
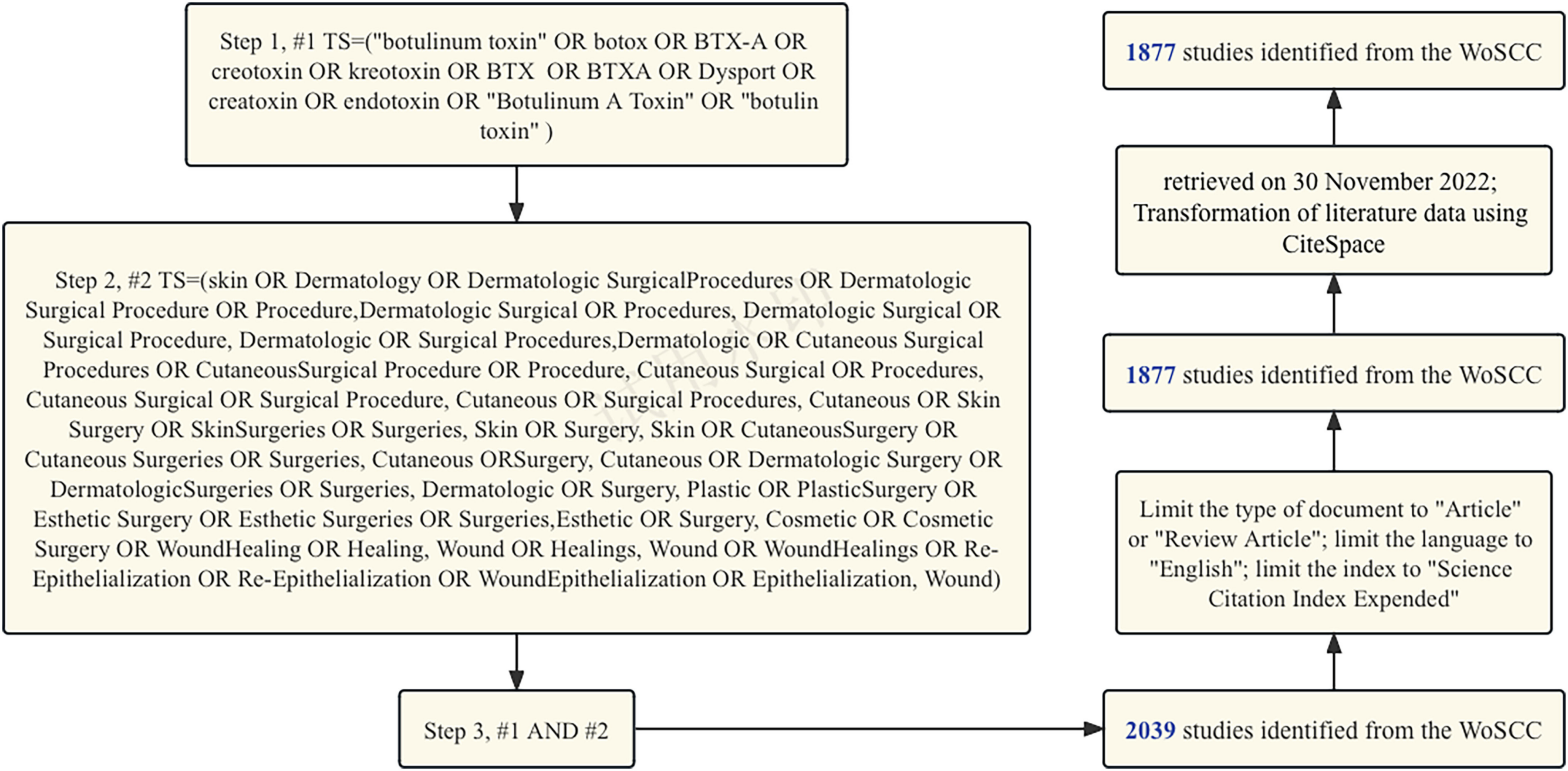
The search flowchart.

## 3 RESULTS

### 3.1 General situation analysis

A total of 1877 documents were collected, with an overall curvilinear upward trend (Figure 2). This suggests that BTX has gradually gained increasing attention from researchers and has become a research hotspot in the field of dermatology.

**FIGURE 2:**
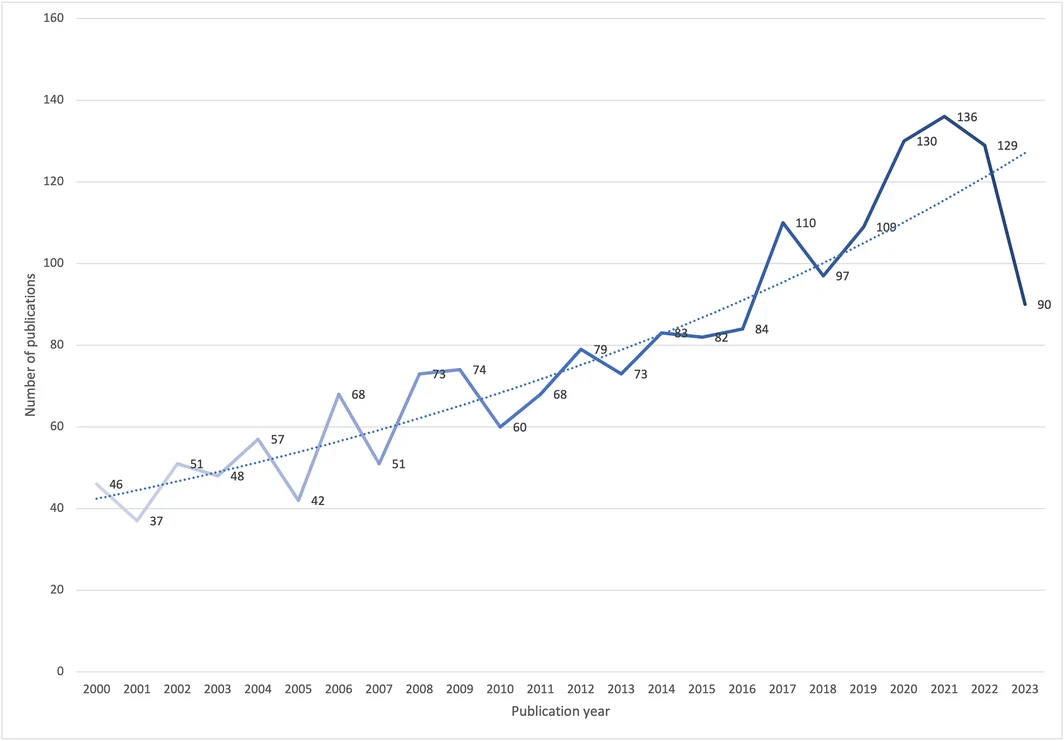
Chronological distribution of publications from 2000 to 2023.

### 3.2 Fund support

A total of 1877 papers were included in this paper, and 510 papers were included with the restriction of “funders,” which represents 510/1877 papers with grant support.

### 3.3 Countries, institutions, and authors co‐operation analysis

Over the past 20 years, publications included in this study have been issued in 43 countries. The merged country network, as created by Scimago Graphica (Figure 3). The United States has the highest publication volume (618 papers), followed by China (231 papers), Germany (162 papers), the United Kingdom (142 papers), and South Korea (126 papers).

**FIGURE 3:**
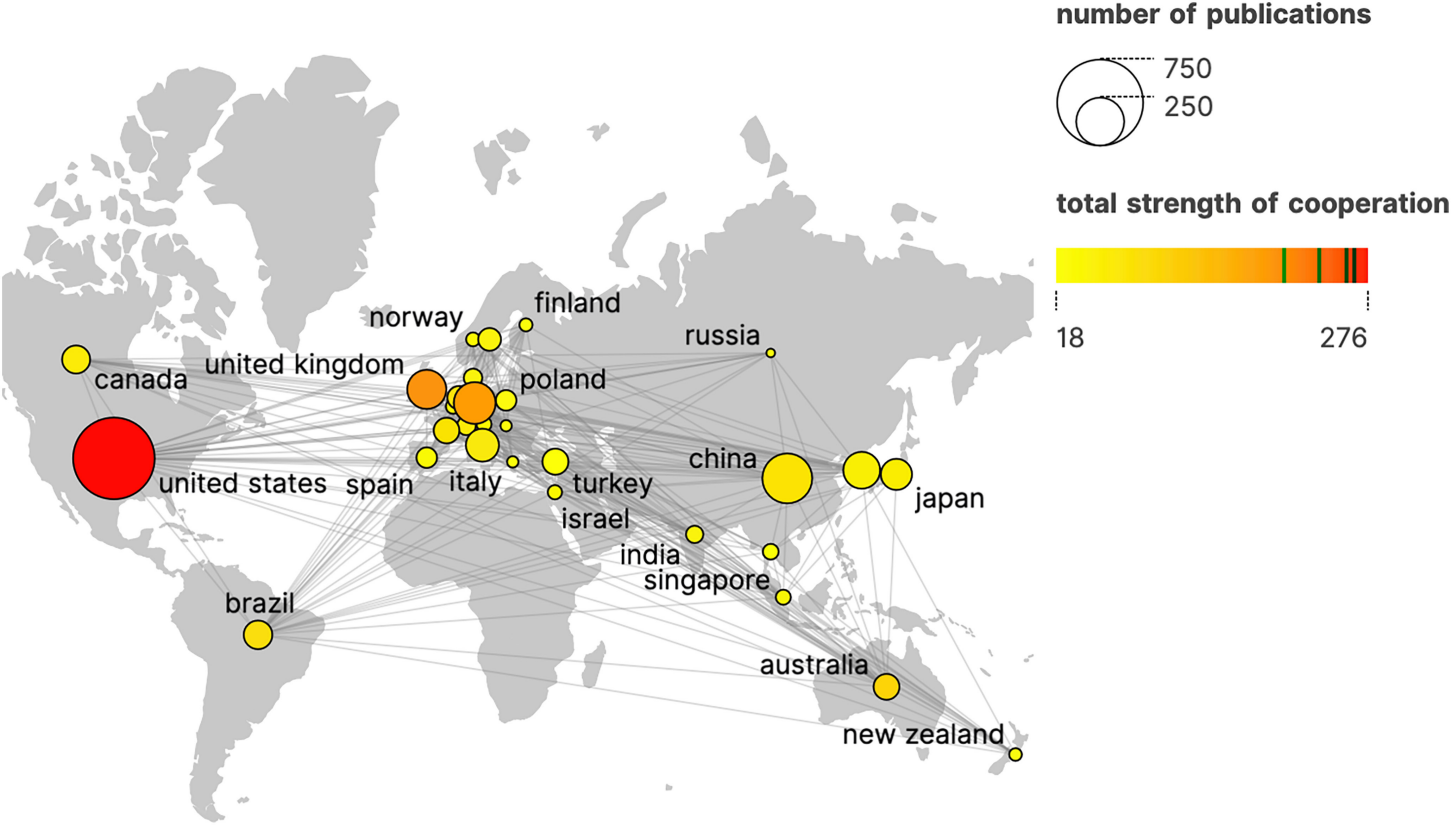
Visual analysis of network co‐occurrence of research cooperation in different countries. (The node size represents the number of publications. The lines between nodes represent the links between the country of publications.)

In terms of major publishing institutions, Yonsei University published the most papers with 28 (Figure 4). Collaboration between the top 10 institutions is mainly regional and international collaboration is still lacking.

**FIGURE 4:**
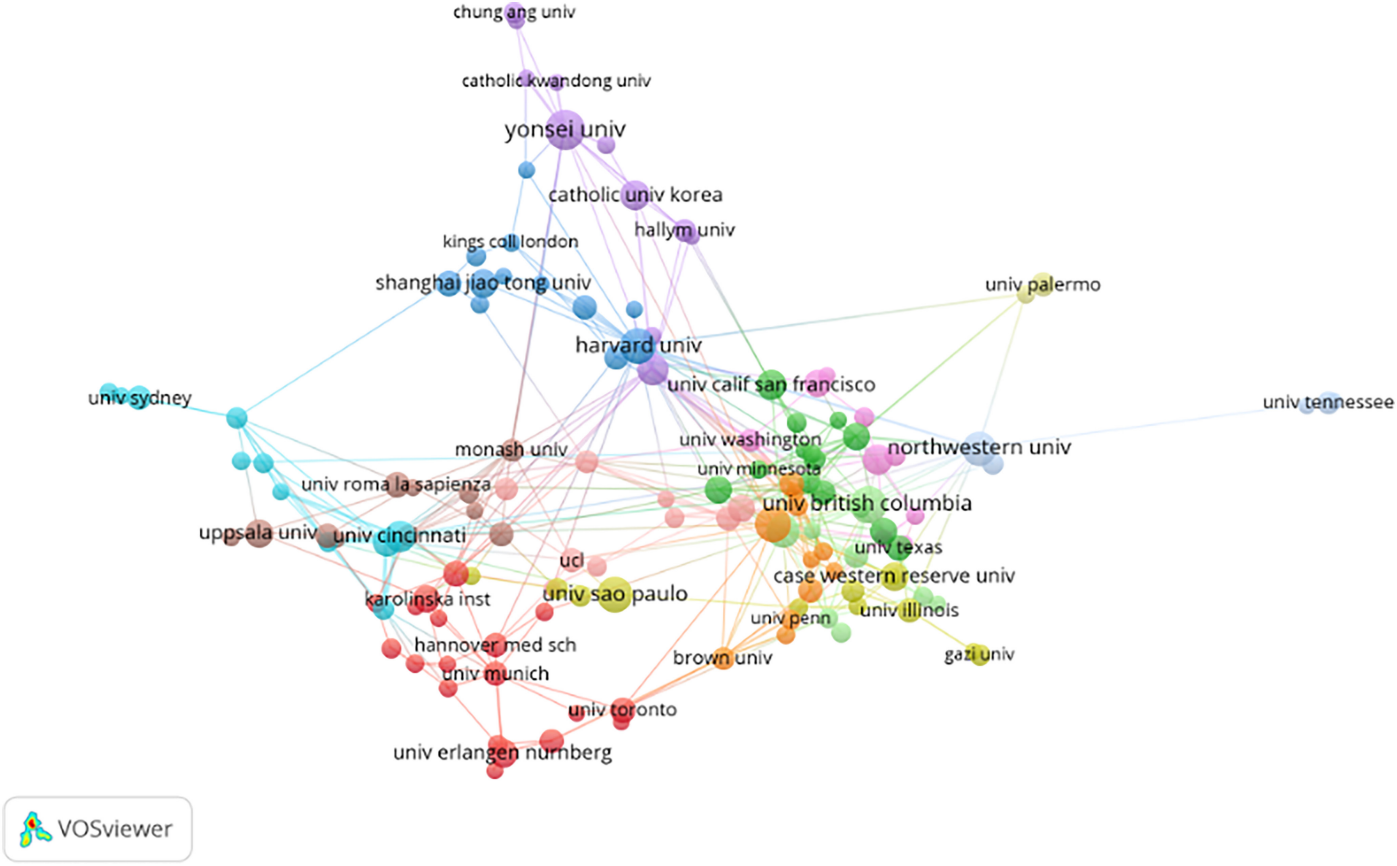
The co‐occurrence map of institutions. (The node size represents the number of publications. The lines between nodes represent the links between the country of publications.)

The analysis of published journals shows that the research publications on the use of BTX in dermatology cover a wide range of fields, including plastic surgery, esthetic dermatology, basic immunology, and so forth, with 21.74% (408 papers) published in the top 10 journals listed in Figure 5.

**FIGURE 5:**
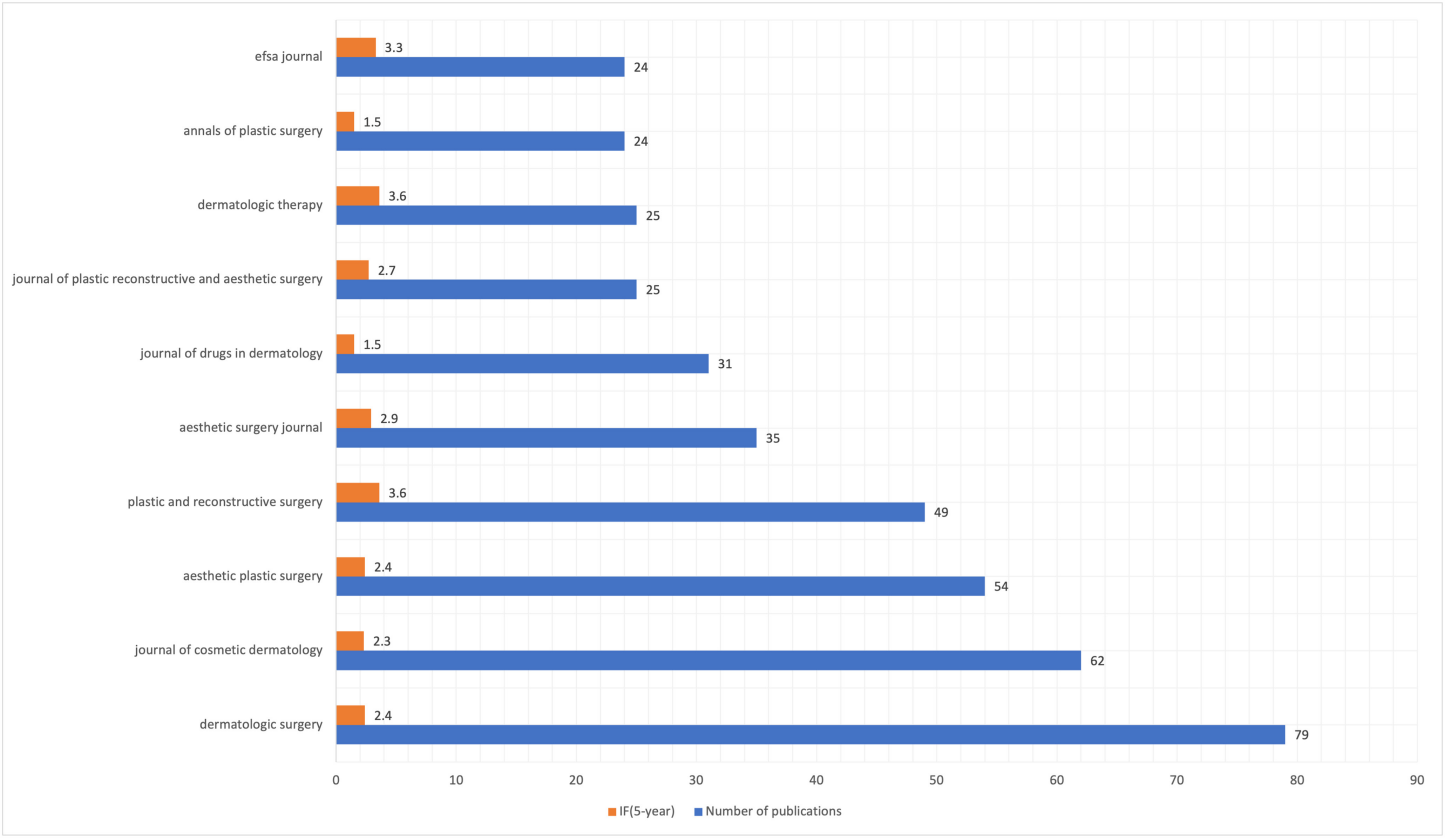
Publication of journal analyzes.

The most prolific author is Lee W Jones with eight papers, followed by Li Jing with six papers and Liu Yang with six papers. Although many authors have published relevant articles, the collaboration among them is scarce.

### 3.4 References co‐operation analysis

CiteSpace was used to generate a co‐citation network diagram (Figure 6) consisting of 1105 co‐cited articles and 3672 links (i.e., the link between individual co‐cited articles). The most cited article was “Botulinum Toxin Off‐Label Use in Dermatology: A Review” by Anna Campanati et al.^12^ It summarized the indications for over‐the‐counter use of botulinum toxin in recent years. Tables 1 and 2 present high‐frequency and high‐centrality articles. (Centrality: The main function of centrality is to measure the importance of the node, the larger the value of centrality, the more important the node is and the more attention the node gets.)^12^, ^13^, ^14^, ^15^, ^16^, ^17^, ^18^, ^19^, ^20^ Generally speaking, literature has a higher citation frequency, and centrality, which represents a greater research significance, influence, and authority, in the field of research.

**FIGURE 6:**
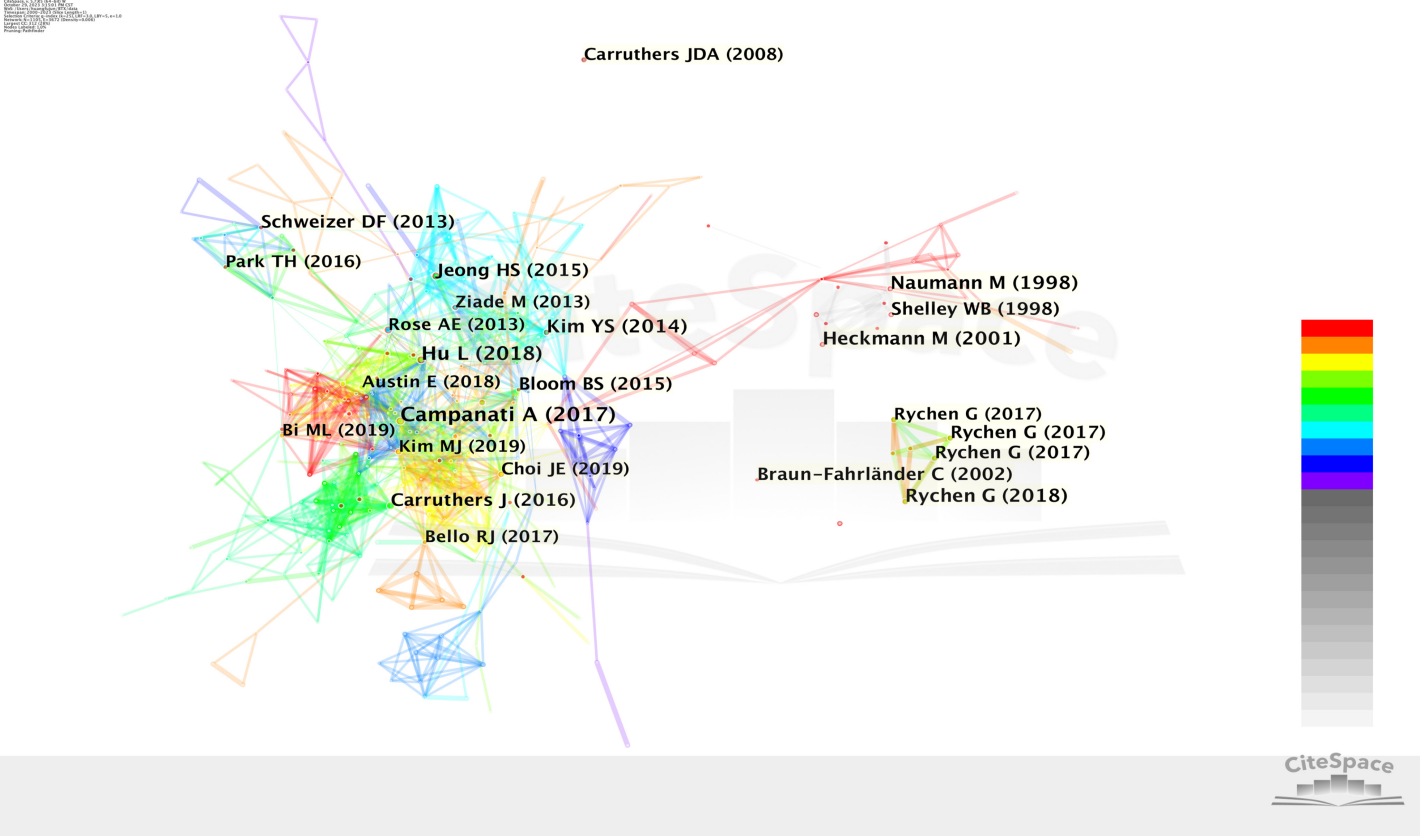
The co‐occurrence map of references.

**TABLE 1:** The top 5 references with cited frequency.

Name	Author	Year	Document title	Source	Citation count
1	Anna Campanati	2017	Botulinum toxin off‐label use in dermatology: a review	Skin Appendage Disor	20
2	Li Hu	2018	Effects of botulinum toxin on improving facial surgical scars: a prospective, split‐scar, double‐blind, randomized controlled trial	Plast Reconstr Surg	17
3	Heckmann M	2001	Botulinum toxin A for axillary hyperhidrosis (excessive sweating)	New Engl J Med	16
4	Guido Rychen	2018	Guidance on the characterisation of microorganisms used as feed additives or as production organisms	EFSA J	16
5	Youn Sung Kim	2014	Early postoperative treatment of thyroidectomy scars using botulinum toxin: a split‐scar, double‐blind randomized controlled trial	Wound Repair Regen	15

**TABLE 2:** The top 5 references with the centrality.

Name	Author	Year	Document title	Source	Centrality
1	Murad Alam	2002	Pain associated with injection of botulinum A exotoxin reconstituted using isotonic sodium chloride with and without preservative: a double‐blind, randomized controlled trial	Arch Dermatol	0.04
2	Anna Campanati	2017	Botulinum toxin off‐label use in dermatology: a review	Skin Appendage Disor	0.03
3	Bradley S Bloom	2015	Impact of intradermal abobotulinumtoxin A on facial erythema of rosacea	Dermatol Surg	0.03
4	Jean Carruthers	2004	Consensus recommendations on the use of botulinum toxin type a in facial esthetics	Plast Reconstr Surg	0.03
5	Amy E Rose	2013	Safety and efficacy of intradermal injection of botulinum toxin for the treatment of oily skin	Dermatol Surg	0.02

### 3.5 Keyword co‐operation analysis

High‐frequency and high‐centrality keywords often reflect the research hotspots of BTX in the field of dermatology. The higher the centrality of a node, the greater its importance in the research field. (However, some nodes with a centrality of 0 may represent the latest hotspots in the field that have not yet been thoroughly researched. Therefore, a centrality of 0 does not entirely negate the importance of the node.) CiteSpace is used to analyze the literature on BTX in dermatology over the past 23 years and generated a co‐occurrence diagram of keywords (Figure 7, comprising 671 nodes (i.e., keywords) and 4985 links). Table 3 presents the top 10 most frequent keywords, while Tables 4 and 5 extract the highest‐ranking keywords related to the indications and adverse reactions of BTX in the field of dermatology, respectively.

**FIGURE 7:**
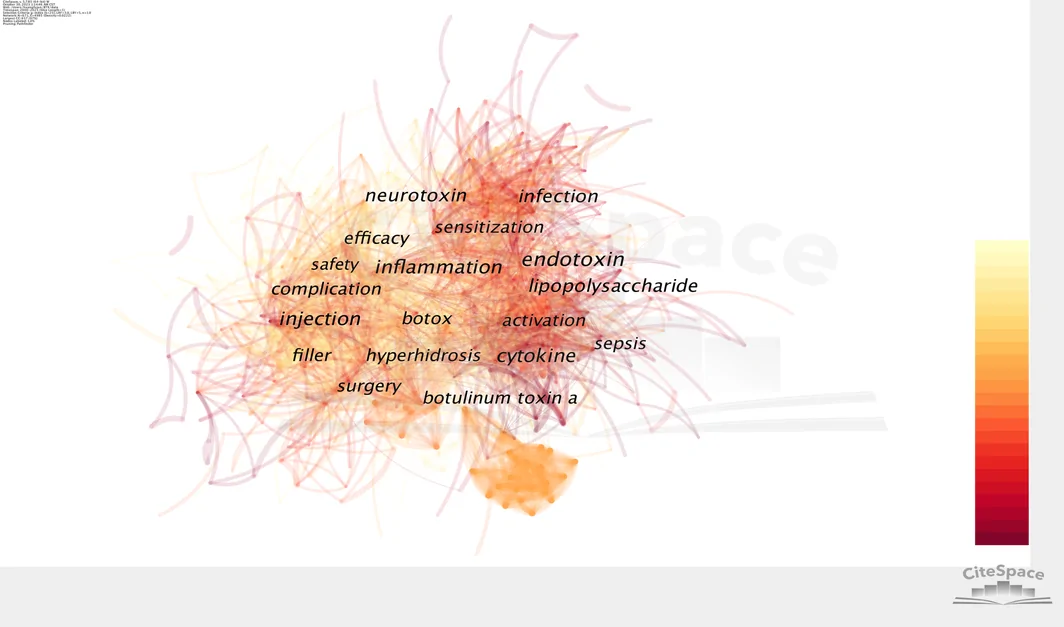
The co‐occurrence map of keywords.

**TABLE 3:** High‐frequency keywords of BTX for dermatology.

Name	High‐frequency keywords	Frequency	Centrality
1	Botulinum toxin A	309	0.06
2	Endotoxin	252	0.13
3	Safety	101	0.03
4	Lipopolysaccharide	99	0.03
5	Botox	91	0.06
6	Cytokine	63	0.02
7	Children	60	0.09
8	Neurotoxin sensitization	42	0.07
9	Mechanism	40	0.04
10	Quality of life	36	0.03

**TABLE 4:** High‐frequency keywords of BTX for dermatology (indications).

Name	High‐frequency keywords	Frequency	Centrality
1	Inflammation	81	0.11
2	Axillary hyperhidrosis	57	0.04
3	Scar after surgery	43	0.07
4	Facial wrinkles	40	0.02
5	Hypertrophic scar	38	0.02
6	Atopic dermatitis	28	0.04
7	Facial rejuvenation	20	0.02
8	Cervical dystonia	16	0.01
9	Palmar hyperhidrosis	11	0.01
10	Esthetic surgery	11	0.01

**TABLE 5:** High‐frequency keywords of BTX for dermatology (adverse reaction).

Name	High‐frequency keywords	Frequency	Centrality
1	Infection	42	0.07
2	Pain	37	0.02
3	Sepsis	35	0.03
4	Allergy	26	0.01
5	Septic shock	11	0.01

Based on the co‐occurrence diagram of keywords, a cluster analysis was conducted using the log‐likelihood ratio to generate a keyword clustering diagram (Figure 8, *Q* = 0.5049, *S* = 0.8254). Generally, the modularity *Q* (*Q*) serves as an indicator for evaluating the effectiveness of network clustering. *Q* >0.3 indicates that the cluster analysis is valid. The weighted mean Silhouette (*S*) is an indicator of the overall structural performance of the map, which is used to evaluate the homogeneity of the clusters, if *S* >0.5 indicates that the clustering is reasonable, and if *S* >0.7 indicates that the clustering is credible. A timezone view was plotted based on the publication dates of high‐frequency keywords (Figure 9): each node in the figure represents a keyword, and the year in which the keyword first appeared in the data analysis is highlighted. Once a keyword appears, it is fixed to the year of its initial appearance and further appearances of the keyword will be cumulatively added. This gives us a clear picture of when each research hotspot started.

**FIGURE 8:**
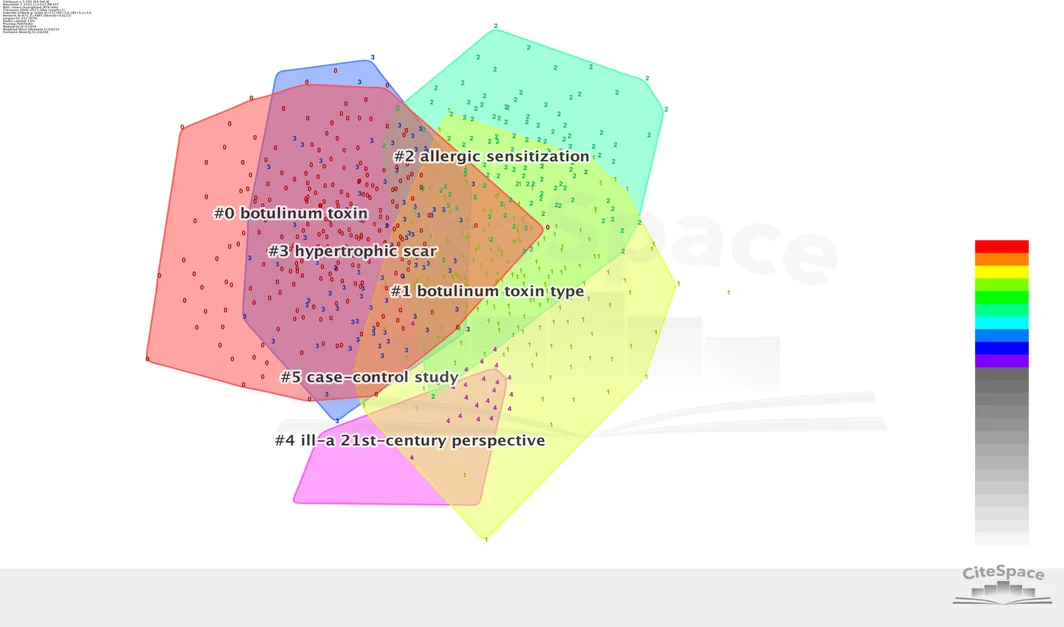
The cluster graph of keywords.

**FIGURE 9:**
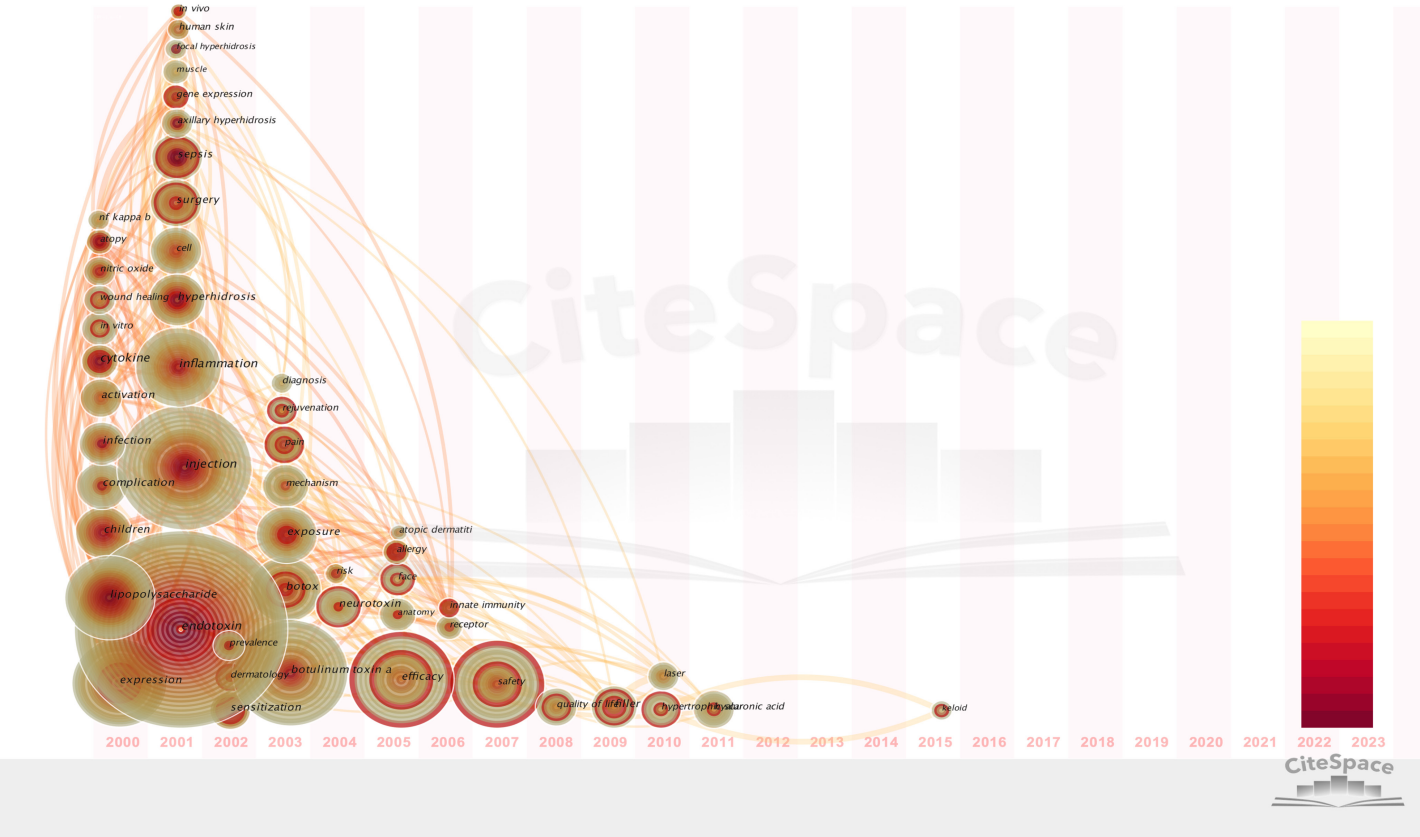
The timezone view of keywords.

“Burstness” has been utilized to detect keywords that have attracted significant attention from scientists at different periods. Figure 10 lists the top 25 keywords, showing the duration and intensity of the top 25 keywords. (In the figure, Year indicates the year in which the document first appeared, Strength indicates the strength of emergence, Begin and End indicate the year in which the keyword started, and ended as a hotspot of academic research (i.e., the red part), the light blue part means that the keyword has not yet appeared, and the dark blue part indicates that the keyword has started to appear.) This effectively reflects the period in which the main research elements and themes emerged and reveals trends.

**FIGURE 10:**
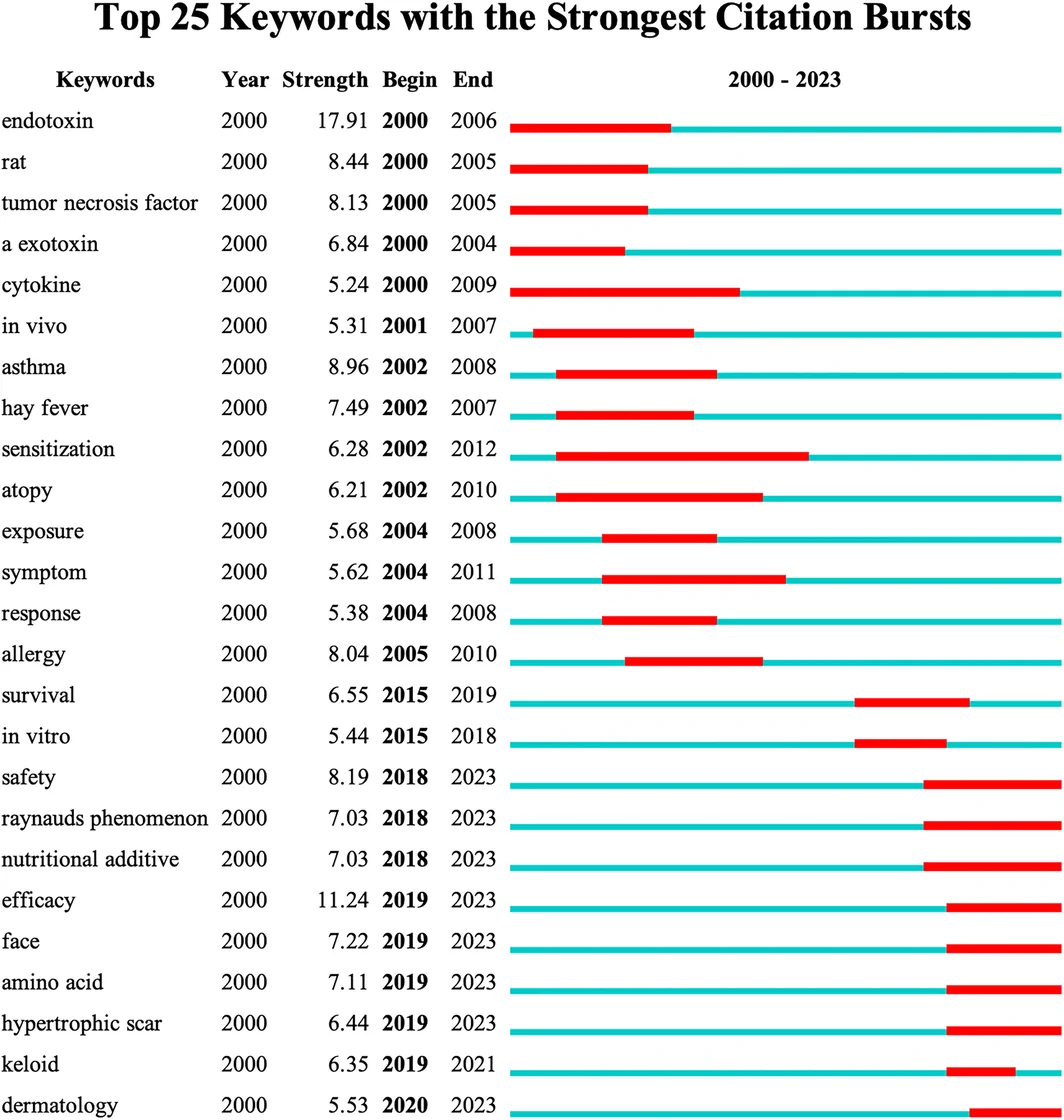
Top 25 keywords with the strongest citation bursts.

## 4 DISCUSSION

Based on keywords analysis, “Burstness” analysis and cluster analysis, we identified the following major research themes.

### 4.1 Classification of BTX

BTX can generally be classified into eight types: A, B, C1, C2, D, E, F, and G. Among them, BTX‐A has the highest toxicity and belongs to the Zn (2^+^)‐dependent family of protein hydrolases. It acts by inhibiting the release of acetylcholine (ACH) from peripheral nerves, leading to anesthesia, nerve blockade, and muscle relaxation. It is commonly used in clinical applications such as facial slimming, calf slimming, and wrinkle reduction. However, the effects of BTX‐A are temporary and reversible. With BTX‐A gradual degradation and inactivation within the nerve endings, the absence of ACH leads to the germination and regeneration of new axons, the original neuromuscular junctions are reactivated, and BTX‐A effect is lost.

The difference between BTX‐A and botulinum toxin type B (BTX‐B) is mainly the difference in amino acid sequence. Both are neurotoxins containing high molecular proteins produced by the bacterium *C. botulinum*, and both can inhibit the release of ACH from nerve endings, which results in muscle relaxation. BTX‐B was approved by the FDA for the treatment of cervical dystonia in December 2000 and was used for various skin and non‐skin diseases.^21^

Botulinum toxin type D (BTX‐D), along with botulinum toxin type C (BTX‐C), is generally used for rodenticides. Recent studies have shown that intradermal injection of BTX‐D can block the autonomic cholinergic synapses in the human body. Compared to BTX‐A, BTX‐D had half the intensity of effect, and a shorter effect duration of about 2 weeks than the former. BTX‐D has a weaker and shorter action, and its therapeutic effect is weaker than that of BTX‐A.^22^

Botulinum toxin type E (BTX‐E) can inhibit the presynaptic release of ACH and block neuromuscular transmission. Similar to BTX‐A and BTX‐B, it has the effect of blocking nerves and relaxing muscles, and so forth.^23^

Currently, there is limited research on botulinum toxin type F (BTX‐F) and botulinum toxin type G (BTX‐G), so we will not elaborate on them further.

In summary, BTX‐A, BTX‐B, and BTX‐E are widely used in dermatology. BTX‐A is often used as a facial‐thinning or leg‐thinning needle to improve muscle hypertrophy due to its muscle paralysis effect. BTX‐B or BTX‐E can be used to reduce wrinkles because of its relaxing effect on muscles.

### 4.2 Clinical applications of BTX

BTX is a superfamily of neurotoxins produced by *C. botulinum*. Its efficacy and safety have been proven in the treatment of facial dynamic wrinkles, muscle hypertrophy in certain areas, excessive muscle tension or spasms in certain areas, and excessive secretion of glands such as sweat glands. From the keywords analysis, cluster analysis, and so forth, we can learn that the mechanism of BTX is relatively clear, and clinical application is widely used.^4^, ^24^ For example: (1) BTX‐A and BTX‐B can reduce neuroinflammation by directly inhibiting mast cell degranulation, significantly inhibit neuropeptides such as substance P and calcitonin gene‐related peptide, which can reduce rosacea, psoriasis, and atopic dermatitis and other skin inflammation^25^, ^26^, ^27^; (2) BTX‐A and BTX‐B can promote the microcirculation of hair follicles, stimulate hair follicle regeneration, and the treatment of hair loss, usually combined with other modalities, the effect is better^28^, ^29^; (3) BTX‐A can prevent the release of ACH from sympathetic nerve fibers and inhibit the secretion of eccrine gland, while ameliorating skin lesions such as Hailey–Hailey, Darier diseases, and so forth^30^, ^31^; (4) BTX‐A can dose‐dependently inhibit the mRNA and protein expression levels of type I collagen and type III collagen induced by TGF‐β1, inhibit the phosphorylation of Smad2/3, and ERK, inhibit the proliferation of hypertrophic scar fibroblasts, and achieve the effects of scar lightening, itching relief, and pain relief^32^; and so on. Meanwhile recent literature reported that BTX can also be used in the treatment of postherpetic neuralgia, but the keyword has not yet been reflected in the visual mapping, indicating that this has not yet been widely used in clinical treatment.^33^

### 4.3 Adverse effects of BTX

In the past 20 years, patients and researchers, alike, have been concerned about the safety of BTX. The most common adverse reactions of BTX are usually local pain, bruising, erythema, swelling, muscle atrophy, and loss of muscle strength due to inappropriate injection depth, injection sites, dosage, or abnormal drug dispersion.^34^, ^35^ Muscle atrophy and loss of muscle strength after BTX injections in the face have been reported to be usually reversible, which can recover from 6 months to 1 year after injection. In severe cases, local infections may occur, or worse, systemic toxicity, anaphylactic shock, infectious shock, and so forth, which is life‐threatening.^36^, ^37^, ^38^

### 4.4 Others

From keywords analysis, “Burstness” analysis, due to the clear mechanism of BTX, there have been fewer studies on its mechanism in recent years. Instead, there have been gradual experimentations with clinical applications, and more and more superscripted indications have been identified. At the same time, the combined effect between BTX and other treatments is gradually being emphasized. For example: (1) Routine BTX injection at the surgical incision site can effectively prevent scar formation^39^; (2) BTX can synergize with hyaluronic acid and autologous fat filling to improve facial width, facial depression, and wrinkles in patients^40^, ^41^, ^42^; (3) BTX combined with platelet‐rich plasma is more effective than monotherapy in treating scarring^43^; (4) BTX combined with photodynamic therapy for Hailey–Hailey disease,^44^ and so on.

## 5 CONCLUSION

BTX is currently widely used in dermatology, mainly in conditions such as hyperhidrosis, wrinkles, muscle hypertrophy, and postoperative scarring, and so forth. We found by analyzing the research regions that the developed countries in Europe and America have the most publications, the most applications, and the highest acceptance. We also found that the safety of BTX as a poison and narcotic drug has been a key concern for a long time. At the same time, by analyzing the high‐frequency keywords, it can be predicted that there is a high interest in how to prevent and treat the adverse effects of BTX, how to explore more undiscovered indications of BTX, and that it may become the next research hotspot or trend.

Despite the contribution of this study, its limitations should be recognized. First, it is important to note that this analysis only incorporates selected articles from the WoSCC database. The analysis was limited to a specific set of keywords, and therefore, a comprehensive analysis of most articles was not feasible. Consequently, the findings presented in this study provide a broad overview and general trends rather than in‐depth insights into each individual publication/keyword. To overcome these limitations and further advance the understanding of BTX treatment for skin diseases, future research should expand the scope of analysis, and stay abreast of the latest. In conclusion, while this study provides a valuable overview of the trends and directions in BTX treatment for skin diseases based on the analysis of articles in the WoSCC database, it is important to recognize its limitations.

In the next step, we will propose to integrate the literature from several databases to analyze and get better data.

## AUTHOR CONTRIBUTIONS

Z.Z and F.H are co‐first authors. Z.Z and F.H conceptualized and designed the study and reviewed the protocol for important intellectual content. Z.Z and F.H drafted the manuscript and collected data. Z.Z and F.H analyzed the data. It has been edited by J.C as native. All authors read and approved the final manuscript.

## CONFLICT OF INTEREST STATEMENT

The authors have no conflict of interest to declare.

## ETHICS STATEMENT

This manuscript did not include any human subjects and animal experiments.

## Data Availability

The data that support the findings of this study are available on request from the corresponding author. The data are not publicly available due to privacy or ethical restrictions.
